# Thrombin-induced microglial activation impairs hippocampal neurogenesis and spatial memory ability in mice

**DOI:** 10.1186/s12993-015-0075-7

**Published:** 2015-09-26

**Authors:** Yuan Yang, Meikui Zhang, Xiaoni Kang, Chen Jiang, Huan Zhang, Pei Wang, Jingjing Li

**Affiliations:** Department of Neurological, Chinese PLA General Hospital, No. 28, Fuxing Road, Haidian District, 100036 Beijing, China; Department of Telemedicine Center, Chinese PLA General Hospital, No. 28, Fuxing Road, Haidian District, 100036 Beijing, China; Department Students Brigade, The Second Military Medical University, No. 800, Xiangyin Road, 200433 Shanghai, China

**Keywords:** Neurogenesis, Intrastriatal hemorrhage, Hirudin, Doublecortin, Microglia/macrophages, Spatial memory deficit

## Abstract

**Background:**

To investigate the effects of microglia/macrophages activation induced by intrastriatal thrombin injection on dentate gyrus neurogenesis and spatial memory ability in mice.

**Methods:**

The male C57BL/6 mice were divided into 4 groups of 10: sham, intracerebral hemorrhage (ICH), ICH + hirudin (thrombin inhibitor), and ICH + indometacin (Indo, an anti-inflammation drug). ICH model was created by intrastriatal thrombin (1U) injection. BrdU (50 mg/kg) was administrated on the same day after surgery for 6 consecutive days. Motor functions were evaluated with rotarod and beam walking tests. The spatial memory deficit was measured with Morris water maze (MWM). Cell quantification was performed for doublecortin (DCX, immature neuron), BrdU (S-phase proliferating cell population) and CD68 (activated microglia/macrophage) immune-reactive cells.

**Results:**

Microglia/macrophages activation induced by intrastriatal thrombin injection reduced hippocampal neurogenesis and impaired spatial memory ability, but did not affect the motor function at 3 and 5 days post-injury. Both hirudin and indometacin reduced microglia/macrophages activation, enhanced hippocampal neurogenesis, and improved spatial memory ability in mice.

**Conclusions:**

Microglia/macrophages activation induced by intrastriatal thrombin injection might be responsible for the spatial memory deficit. Targeting both thrombin and inflammation systems in acute phase of ICH might be important in alleviating the significant spatial memory deficits.

## Background

Learning and memory deficits are prevalent in patients with striatum ICH [1, 2]. The hematoma in most of the patients is locally limited to the striatum without direct contact with hippocampus; therefore, the cognitive deficits following striatum ICH might not be caused by detectable brain lesions. The thrombin induced blood brain barrier (BBB) disruption, cerebral edema, and central nervous system (CNS) inflammation are major secondary injuries of ICH and might be therapeutic targets in medical intervention [1–3].

The inflammation after ICH is an important second injury [3, 4]. Studies indicate that inhibition of the inflammatory elements in the brain, such as microglia, infiltrating peripheral leukocytes and pro-inflammatory cytokines, improves neurological scores and decreases hemorrhage volume in animal models [5, 6]. Among these elements of brain inflammation, microglia activation might play a major role in the secondary damage of ICH [7]. It has been reported that the increased density of activated microglia cells contribute to neuronal damage in both acute diseases (e.g., acute stroke and acute infection) and chronic neurodegenerative diseases (e.g., Alzheimer’s disease, Parkinson’s disease and multiple sclerosis) [8]. Moreover, studies have shown that thrombin or autologous blood microinjection into the striatum of adult rats induced neuronal death and microglial activation around or adjacent to the injection site [9]. The microglia in tissues surrounding the hematoma increased within 1 h of post-injury, remained elevated for several days, and subsequently decreased over several weeks [10].

The impact of inflammation, especially microglia activation, on learning and memory after ICH remains unclear.

Adult hippocampal neurogenesis, which is represented by DCX^+^ and DCX/BrdU^+^ cells in the dentate gyrus, is closely related to hippocampal plasticity and is implicated in spatial memory formation and processing [11, 12]. Damage to the neurogenesis in the dentate gyrus impairs the performance in water maze tasks, and the severity of the impairment is proportional to the extent of damage specific to the granule cell progenitor population [13, 14]. Some studies have shown that the water maze performance was not affected by adult neurogenesis impairment; however, the test protocols used in these studies were considerably different compared to other studies [15].

Therefore, we explored the correlation among the microglia/macrophages activation, the neurogenesis in dentate gyrus of hippocampus and the MWM performance of mice after ICH.

Most ICH models target the striatum since it is one of the most common sites of ICH in humans [16]. In the striatum ICH model however, little attention has been paid to the function of distal brain regions, such as the neurogenesis in dentate gyrus. The involvement of inflammation and microglia activation in spatial memory deficits after striatum ICH could be supported by some indirect evidence. For example, the numbers of activated microglia and new-born neurons (BrdU^+^/NeuN^+^ cells) are negatively correlated in a model of intraperitoneal injection of LPS [17]. The number of CD68 (ED-1) positive microglia is also negatively correlated with neurogenesis during inflammation induced by cranial irradiation [18]. Furthermore, one in vivo study demonstrates that intrastriatal thrombin injection activates microglial cells in the midbrain and causes dopaminergic neuron death [19]. Along the rostral to caudal axis of the brain, the substantial nigra is located further away from the thrombin injection site than hippocampus. The damaging effects of microglia during stress or diseases are usually mediated through soluble factors, such as pro-inflammatory cytokines or reactive oxygen species (ROS) [7]. Therefore, it is possible that the hippocampal neurogenesis might be affected by ICH caused by intrastriatal injection of thrombin.

In this study we will investigate the microglia activation in subventricular zone (SVZ) and subgranular zone (SGZ) after the intrastriatal thrombin injection and explore the effects of anti-inflammation drugs on microglia activation and neurogenesis impairment.

Two related issues are addressed in this study. Firstly, if hippocampal neurogenesis was affected by inflammation, the source of inflammation would be from the intrastriatal thrombin injection. Therefore the inflammation of the brain regions surrounding the injection tract should be observed together with the distant region, such as hippocampus, to confirm that the inflammation was originated from the thrombin injection.

The second one is that the MWM performance might be affected by the ICH induced motor or coordination deficit. The rotarod test is used to investigate neurological functions, assess motor coordination and balance, and to explore brain injury such as stroke in mice [20]; while beam walking assay is used to detect motor deficits in both rats and mice [21, 22]. These two behavioral tests were selected to measure the motor and coordination deficits in the thrombin injured mice.

## Methods

**Fig. 1:**
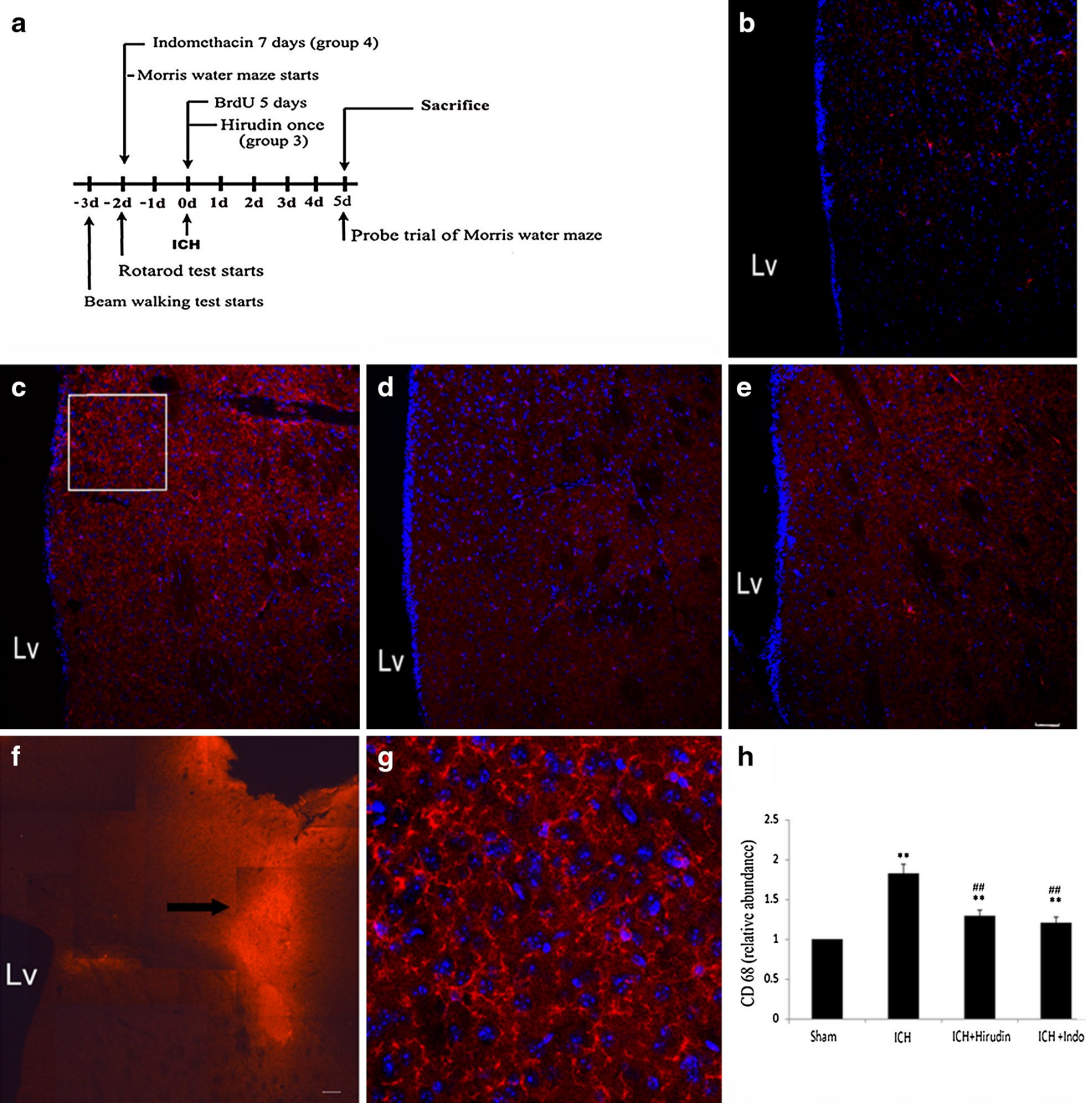
CD68 staining of activated microglia/microphage cells in the SVZ. **a** The schematic diagram of the animal use and the timeline of experiments. The training or adaptive time for the rotarod and Morris water maze group was 2 days, and for beam walking was 3 days. The mice were tested at day 1, 2, and 3 for water maze, at day 5 for the probe trial, and at day 1, 3, 5 for rotarod and beam walking tests. The animals were sacrificed at day 5. **b**–**e** The representative CD68 staining and DAPI nucleus staining near thrombin (1U) injection site in sham (**b**), ICH (**c**), ICH + hirudin (**d**), and ICH + indo (**e**) groups. **f** The CD68 staining at the injection tract. The right lateral ventricle (LV) and the right SVZ were located in the vicinity of the thrombin infusion tract. SGZ of hippocampus was distal to the injection tract and could not be seen on the graph. **g** The amplified square area in **c**. **h** The CD68 abundance was normalized to sham group. After the treatment of thrombin (1U), the CD68 signal of activated microglia cells in the SVZ area increased significantly in comparison to sham group, indicating an increase in the number and/or hypertrophy of microglia cells. The administration of hirudin at 20 min after the injection of thrombion (1U) significantly inhibited the increase of CD68 abundance. The administration of indo also significantly decreased the CD68 abundance. **p < 0.01, compared with sham; ^##^p < 0.01, compared with ICH group. *LV* lateral ventricle. *Blue* DAPI; *red* CD68; “” transplantation tract, n = 10 mice/group; *scale bar* 50 µm

All animal experiments were performed in accordance with the guidelines approved by the ethics committee of Chinese PLA General Hospital. The animal use and the timeline of experiments were demonstrated in Fig. 1a. We used two cohorts of male C57BL/6 mice, weighting 24 ± 2 g, 9–10 weeks of age. The first cohort contained 60 and the second cohort contained 40 animals. Each cohort was divided into 4 groups equally. The groups were as the follows: Group 1 (Sham): mice received intrastriatal saline injection; Group 2 (ICH): mice received intrastriatal thrombin (1U) injection. Group 3 (ICH + hirudin): mice received intrastriatal thrombin (1U) injection and hirudin (a specific inhibitor of thrombin, (1U) treatment. The hirudin was dissolved in 2 μL PBS and infused (1 μL/min) into the same location 20 min after ICH.

Group 4 (ICH + indomethacin): mice received intrastriatal thrombin (1U) injection and indomethacin (an anti-inflammation drug) treatment. Indomethacin (Sigma-Aldrich, St Louis, MO, USA) was dissolved in strawberry flavored milk at a concentration of 0.2 mg/mL. Mice were allowed free access to the milk and consumed the flavored milk at a dose of (1 mg/kg/day). The administration of indomethacin started 2 days before the ICH model was made, and continued until the animals were sacrificed for a total of 8 days.

The mice in cohort 1 were used for the water maze test, immunohistochemistry assay of neurogenesis and microglia/macrophage activation. The mice in the cohort 2 were used for the motor function test.

### ICH model

Mice were anesthetized using a mixture of ketamine/xylazine. Animals were placed in a stereotaxic frame using modified ear-bars fitted with blunt rubber ends designed for mice. A midline scalp incision was made, and a hole was drilled in the right skull (1.7 mm lateral to the midline, 0.02 mm anterior to the bregma). A 26-gauge needle was attached to the syringe and stereotaxically advanced into the striatum 3.0 mm below the surface of the drilled hole in the skull. Thrombin (Sigma, St. Louis, MO, USA; 1 U in 4 μL 0.9 % NaCl) was injected over a 2-min period, and the needle remained at the injection site for an additional 5 min. After slowly withdrawing the needle, the incision was closed, and animals were allowed to recover in a warm, non-stimulating environment with free access to food and water.

### Rotarod test

The mice were trained for 2 days on a rotarod (TSE System, Bad Homburg, Germany) before thrombin injection with 3 consecutive trials per day. Each trial consisted of two 2-min sessions: the rotarod accelerated up to 40 r.p.m during the first 2-min session, and the rotarod ran constantly at 40 r.p.m during the second 2-min session. The time the mice walked on the rotarod when it was running at 40 r.p.m was recorded. At 1, 3 and 5 days after the ICH model was made, the mice walked on the rotarod for a maximum of 120 s per trial for three consecutive trials.

### Beam walking

The beam was made of wood (8 mm in diameter, 80 cm in length) and elevated 30 cm above the bench by metal supports. The mice were trained for 3 days before the ICH surgery. The training protocol was in accordance with the methods published by Fleming [23]. During the training sessions, mice were allowed to walk from one end of the beam to the other end. A home box was held by the experimenter in front of the mice and moved along with the mice to make them think that there would be a home box that could be reached soon. At 1, 3 and 5 days after the thrombin injection, three trials were performed for each mouse per day. In the test session, the mice were placed on the beam at one end and allowed to walk to the other end where a home box was located. The maximum duration of the test session was 60 s. If the mice fell before the 60 s ended, they were returned to the location where they fell from and continued the test until the 60 s ended. The time spent on beam, the number of foot slips (one or more limbs slipped from the beam) and the number of falls was recorded.

### Morris water maze test

The Morris water maze protocol was adapted from published procedures with minor modifications [24]. Briefly, before thrombin injection (day 0), the mice received habituation trials for 2 consecutive days (day −2 and day −1), in which the preferences between quadrants in the different experimental groups were eliminated. The mice were then trained to find a hidden platform over the next 3 consecutive days, with 4 trials/day, 60 s per trial, and 20 s on the platform. If an animal failed to find the platform within 60 s, it was guided to the platform and kept on it for 20 s. In general, two trials were given in the morning with an interval of 40–60 min, and the other 2 trials were given in the afternoon with the same intervals. After 2 days rest, the animals received a probe trial on the 5th day. During the probe trial, the platform was removed for the assessment of platform quadrant preference.

In the water maze tests, the mean escape latency (two-way repeated measures ANOVA), swimming speed (two-way repeated measures ANOVA), and preference for the P quadrant (one-way ANOVA) were recorded and analyzed.

### 5-Bromo-2-deoxyuridine treatment

5-Bromo-2-deoxyuridine (BrdU) was purchased from Sigma (Sigma-Aldrich, St Louis, MO, USA). Animals received daily i.p. injections of BrdU (50 mg/kg, dissolved in 0.9 % NaCl) at a concentration of 10 mg/mL. The BrdU administration started at 2 h after ICH surgery for 6 consecutive days, until the mice were sacrificed.

### Immunohistochemistry and cell count

Mice were anesthetized and transcardially perfused with PBS, followed by cold 4 % paraformaldehyde (PFA). The brains were removed, and post-fixed in 4 % PFA for 24 h. The brains were stored in 30 % sucrose/PBS at 4 °C. All brains were sectioned using vibrating microtome at a thickness of 40-μm. Free-floating sections of the entire brain were collected.

For analysis of immunohistochemistry (IHC) labeled cell number, brain sections were incubated in 0.3 % triton-100 PBS, and blocked with 2 % donkey serum. Sections were incubated overnight with monoclonal antibodies for doublecortin (1:500, Santa Cruz Biotechnology, Santa Cruz, CA, USA) to label newborn neurons, BrdU (1:1000, Becton–Dickinson, San Jose, CA, USA) to label the cells in the S-phase of cell cycle, and CD68 (1:500, Abcam, New territories, HK, China) to label the activated microglia/macrophages cells. The sections were incubated with FITC or Cy3 conjugated secondary antibodies (1:300; Immune-Jackson, Inc., CA, USA) for 2 h at room temperature, followed by DAPI treatment for 20 min.

After being washed and mounted onto slides, the injection tract was firstly examined using a Nikon TE-2000U fluorescence microscope equipped with SPOT capturing software.

For DCX^+^ and DCX^+^/BrdU^+^ cell counts, the digital images and DAPI (blue color) fluorescence were acquired on a confocal Laser Scanning Microscope (Leica, Biberach 88400, Germany) at 200× magnification. The confocal settings, such as gain and offset, were designed to ensure all pixels of all of the selected sections were within the photomultiplier detection range. The setting was maintained to ensure all images were collected with the same parameters. The areas near the SVZ and the SGZ was outlined (within 400 µm to SVZ, 100 µm to SGZ). Quantification was performed at the ipsilateral (injured) SGZ, 1:6 series of the coronal sections containing SGZ were selected for quantification. Only the newborn neurons (DCX and BrdU double positive cells) that were located at the SGZ in dentate gyrus were included and were counted as previously described [25]. The estimated cell numbers were determined using the di-sector method based on the average object diameter and section thickness [26]. All analyses were performed by investigators blinded to the sample identity and treatment groups.

### Fluorescence abundance quantitation

Activated microglia/macrophages were stained with CD68 and the fluorescence abundance of CD68 was quantified. The digital images of CD68 (red color) and DAPI (blue color) fluorescence were acquired on a confocal Laser Scanning Microscope (Leica, Biberach 88400, Germany) at 200× magnification. The areas near the SVZ and the SGZ was outlined. The Image J software was used to analyze pixel abundance. In each image, the background value was set using the ‘‘threshold’’ function of the software to exclude all pixels surrounding the positive stained signals. The positive pixels above the background of the outlined region were recorded and normalized to sham group.

### Statistics

The data of cell counting were expressed as mean ± SEM. After being normalized to the Sham group, the relative fluorescence abundance was expressed as mean ± SEM. These data were analyzed to ensure normal distribution. The comparisons were analyzed by one-way analysis of variance (one-way ANOVA) and Bonferroni–Dunnett corrections using SPSS 10.0. All behavior test data were analyzed by two-way repeated-measures analysis of variance with Student–Newman–Keuls post hoc tests using SPSS 10.0. The level of significance of all comparisons was set at p < 0.05.

## Results

### Thrombin induced microglia/macrophages activation in the areas near and distant to the injection site

CD68^+^ cells were seen at 5 days after saline or thrombin injection in all of the groups (Fig. 1b–e). Compared with the sham group (Fig. 1b, h), activated microglia/macrophages significantly increased in the brain tissues (within 400 µm to SVZ, 100 µm to SGZ) in the ICH group (Fig. 1c, h), suggesting thrombin injection induced a significant increase in the number and/or hypertrophy of activated microglia/macrophages cells in the injection site and nearby brain area. The square area in Fig. 1c was amplified in Fig. 1g to demonstrate the ramified morphology of CD 68^+^ microglia/macrophages.

Strong CD68 signals, a marker of activated microglia/macrophages, could be detected along the injection tract (Fig. 1f). Because the injection site was adjacent to the lateral ventricle (LV), the coronal sections containing SVZ were selected to represent the brain areas near the injection site (Fig. 1f).

**Fig. 2:**
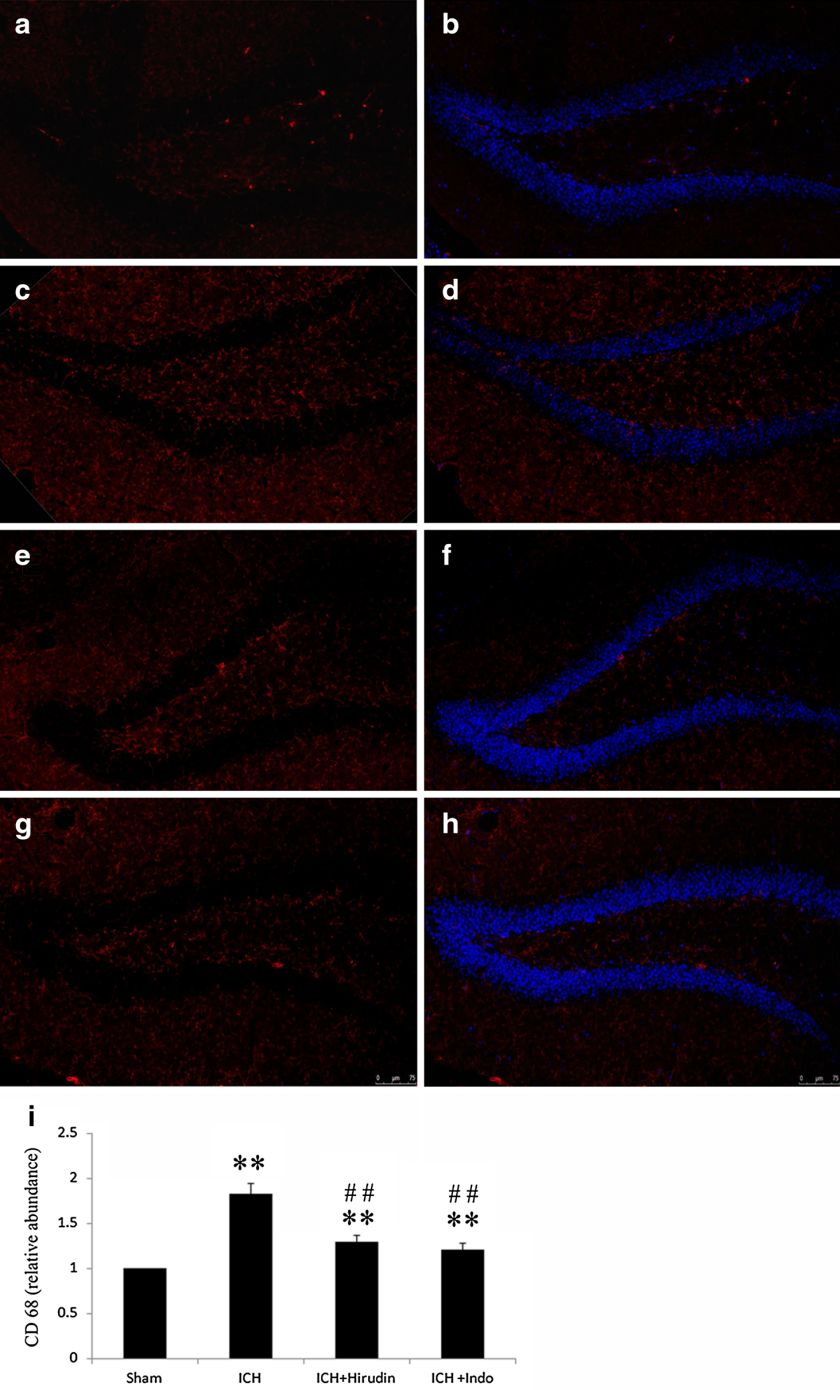
The effect of anti-inflammation drugs on thrombin induced microglia proliferation in the DG of hippocampus. **a** The CD68 immuno-staining in sham mice. **c** The CD68 staining of microglia cells in the DG in ICH group. **e** The CD68 immuno-staining in the “thrombin + hirudin” group. **g** The CD68 staining in “thrombin + indo” group. **b**, **d**, **f**, **h** DAPI staining of **a**, **c**, **e**, **g**. **i** Compared with the CD68 pixel abundance of sham, the injection of thrombin in the striatum leads to increased microglia cells in the hippocampus. Hirudin (the specific inhibitor of thrombin) significantly decreased the microglia density as compared with ICH group. Indo (the anti-inflammation drug) also decreased the CD68 pixel abundance in the hippocampus. n = 10 mice/group. **p < 0.01, compared with sham, ^##^p < 0.01, compared with ICH group, *blue* DAPI; *red* CD68; *scale bar* 50 µm

In our experiments, SGZ of dentate gyrus was distal to the injection site and did not show visible tissue damages or hematoma. However, increased microglia/macrophages activation was found in all of the coronal sections containing DG (Fig. 2c, d) and the pixel abundance of activated microglia/macrophages measured in the DG increased significantly compared with the sham group (Fig. 2a, b).

### The activation of microglia/macrophages was ameliorated by thrombin inhibitor and anti-inflammation drug

To investigate whether the activation of microglia/macrophages were caused by thrombin injection and were involved in brain inflammation, we treated the mice with either hirudin or indometacin. Hirudin treatment significantly decreased CD68 immunoreactivity at SVZ, which was in the vicinity of the thrombin injection site, compared with ICH group (Fig. 1d, h). Meanwhile, hirudin decreased the CD68^+^ microglia/macrophages cells at the DG, which was distal to the injection site, at 7 days after the ICH surgery (Fig. 2e, f). The treatment of indomethacin, one of the nonsteroidal anti-inflammatory drugs (NSAIDS), suppressed microglia/macrophages activation at both the injection site (Fig. 1e, h) and in the DG region of hippocampus (Fig. 2g, h) (**p < 0.01, n = 10). Although hirudin and indomethacin significantly reduced the thrombin induced microglia/macrophages activation, the treatments failed to decrease the microglia/macrophages activation to the sham level. Moreover, there was no difference between the two treatment groups (Figs. 1h, 2i).

### Thrombin inhibitor and anti-inflammation treatment improved the survival of DCX^+^ cells, and DCX^+^ BrdU^+^ neurons in DG of hippocampus

**Fig. 3:**
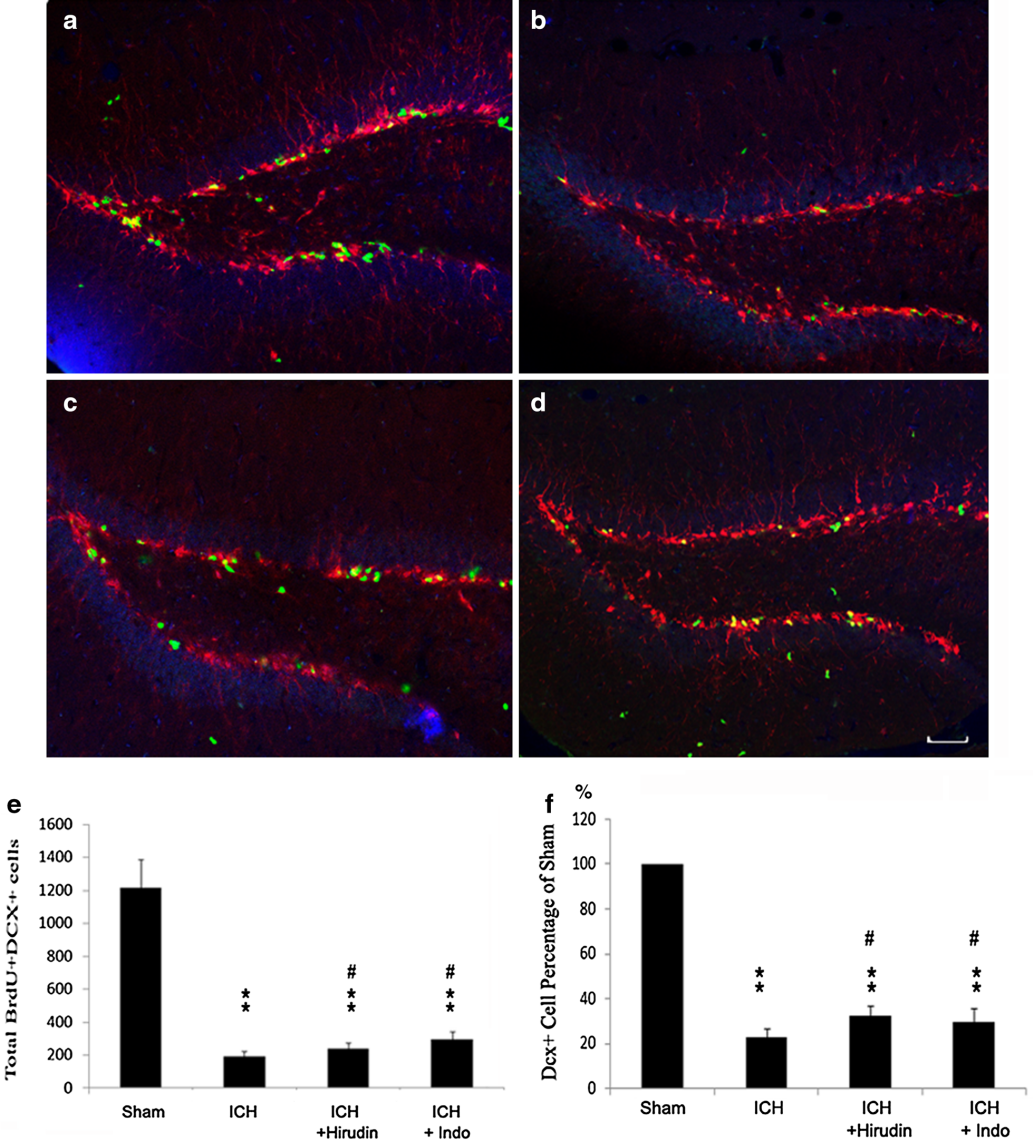
Cell proliferation measured as DCX^+^/BrdU^+^ staining in the dentate gyrus of hippocampus. **a** Sham mice. **b** 1U thrombin injured mice. The brain injury caused by thrombin decreased the DCX^+^/Brdu^+^ cells in the mouse dentate gyrus of dorsal hippocampus. **c** Mice received intrastriatal injection of thrombin and treated with indo. **d** Mice received intrastriatal injection of thrombin and treated with hirudin (a thrombin inhibitor). **e** Total number of BrdU^+^/DCX^+^ cells counted in the DG of all groups. Compared with the sham group, BrdU^+^/DCX^+^ cell number was significantly decreased by 1U thrombin injection, and was reversed by hirudin administration. **f** 1U thrombin injection significantly decreased the DCX^+^ cells in SGZ region as compared with sham. Hirudin and indomethacin treatment after 1U thrombin injection significantly ameliorated the DCX positive cell losses. **p < 0.01, compared with sham; ^#^p < 0.05, compared with ICH group. *Blue* DAPI; *green* BrdU; *red* DCX; n = 10 mice/group; *scale bar* 50 µm

DCX^+^/BrdU^+^ double labeled cells represented the newborn neurons in the SGZ of DG [25]. Compared with sham (Fig. 3a), DCX^+^/BrdU^+^ cells in DG was significantly reduced by 1U thrombin injection (sham: 1213 ± 174 vs. ICH: 192 ± 32) (Fig. 3b). After the treatment of hirudin (Fig. 3c) or indo (Fig. 3d), the DCX^+^/BrdU^+^ cells in DG increased significantly in comparison with ICH group (hirudin: 240 ± 34 and indo: 295 ± 45), respectively (Fig. 3e). However, hirudin and indo treatments did not recover DCX^+^/BrdU^+^ cells to the sham group level.

Compared with the sham group (Fig. 3f); 1U thrombin injection significantly decreased the DCX^+^ cells in SGZ region to 22.43 ± 3.97 % (Fig. 3f). Furthermore, compared with ICH group, hirudin treatment after 1U thrombin injection significantly ameliorated the DCX positive cell loss to 32.21 ± 4.97 % (Fig. 3f) (p < 0.01); whereas the indomethacin treatment significantly ameliorated the DCX positive cell loss to 29.38 ± 6.34 % (p < 0.05).

### The effects of thrombin injection and drug treatments on the motor function of mice

To exclude the possibility that the spatial memory test might be interfered by the thrombin induced motor function deficit, we performed the rotarod fatigue test and beam walking test. We found that after the training sessions all of the mice were able to stay on the running rotarod for 2 min during acceleration and 2 min during a constant speed of 40 rpm. At 1, 3 and 5 days after the thrombin injection into the striatum, all the animals could maintain 120 s on the rotarod when it was running at 40 rpm. That means at 1–5 days after thrombin injection neither the ICH injury, nor the drugs (hirudin and indo) changed the time of the mice spent on the rotarod.

**Fig. 4:**
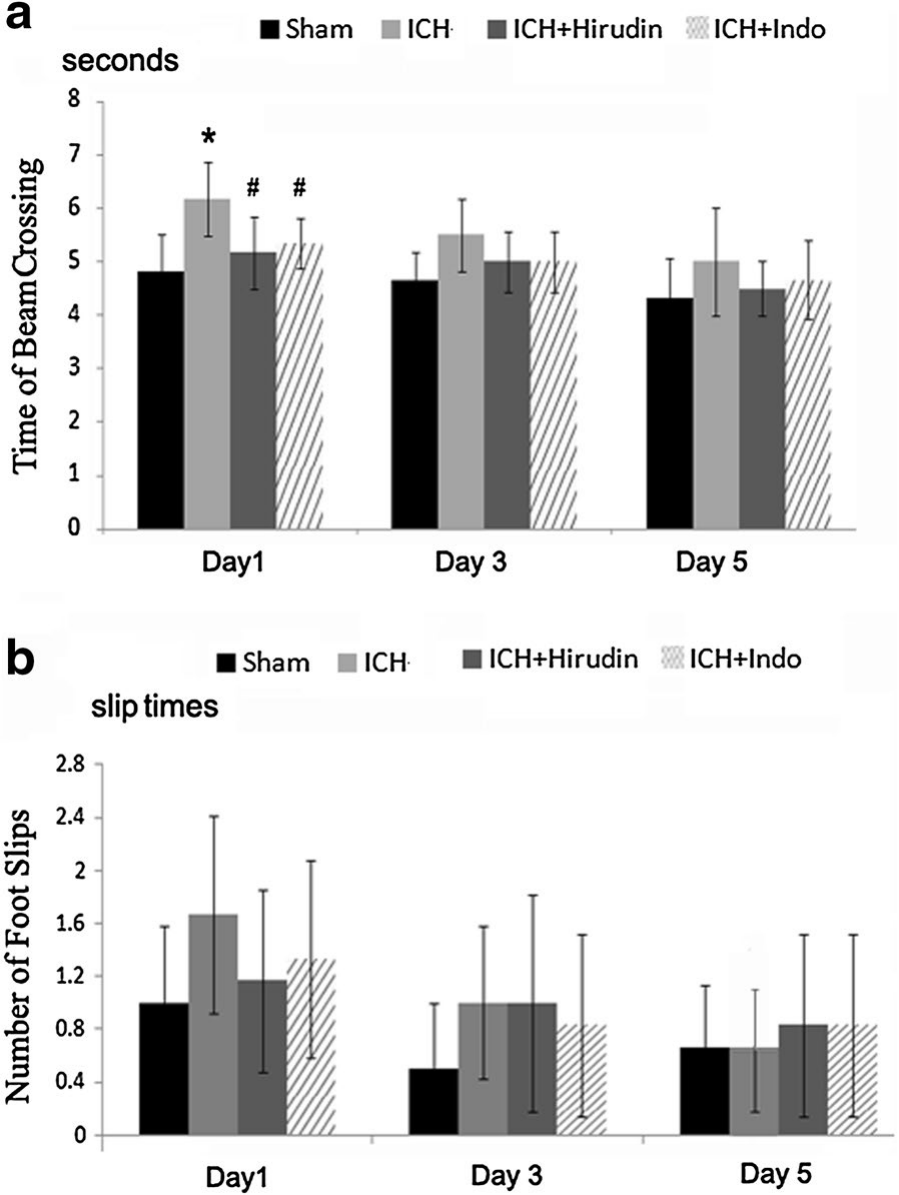
Thrombin injection and drug treatments did not impair the motor function of the mice. **a** At 1 day after ICH, the thrombin injected mice showed significantly reduced speed in the walking beam test. The treatment of hirudin and indo decreased the time spent by the mice in finishing the task. At day 3 and day 5, the walking beam test did not show any difference among all of the groups. **b** No difference of foot slips among all the groups were found at 1, 3, 5 days after the thrombin injection

In beam walking test, all of the tested mice did not fall from the beam and finished the travel from one end of the beam to the other in 4–7 s. Only at 1 day after the thrombin injection, the ICH group showed a slight but significant increase in the time to finish the test as compared with sham group. At 3 and 5 days after the injury, no difference among all the groups was observed (Fig. 4a). As for the number of foot slips, the mice showed 0–2 times of foot slip during the walking on the beam and we did not find any difference among the tested groups at all of the 3 observing time points (Fig. 4b).

### Thrombin inhibitor and anti-inflammation treatments improved the learning and memory ability after thrombin induced brain injury

**Fig. 5:**
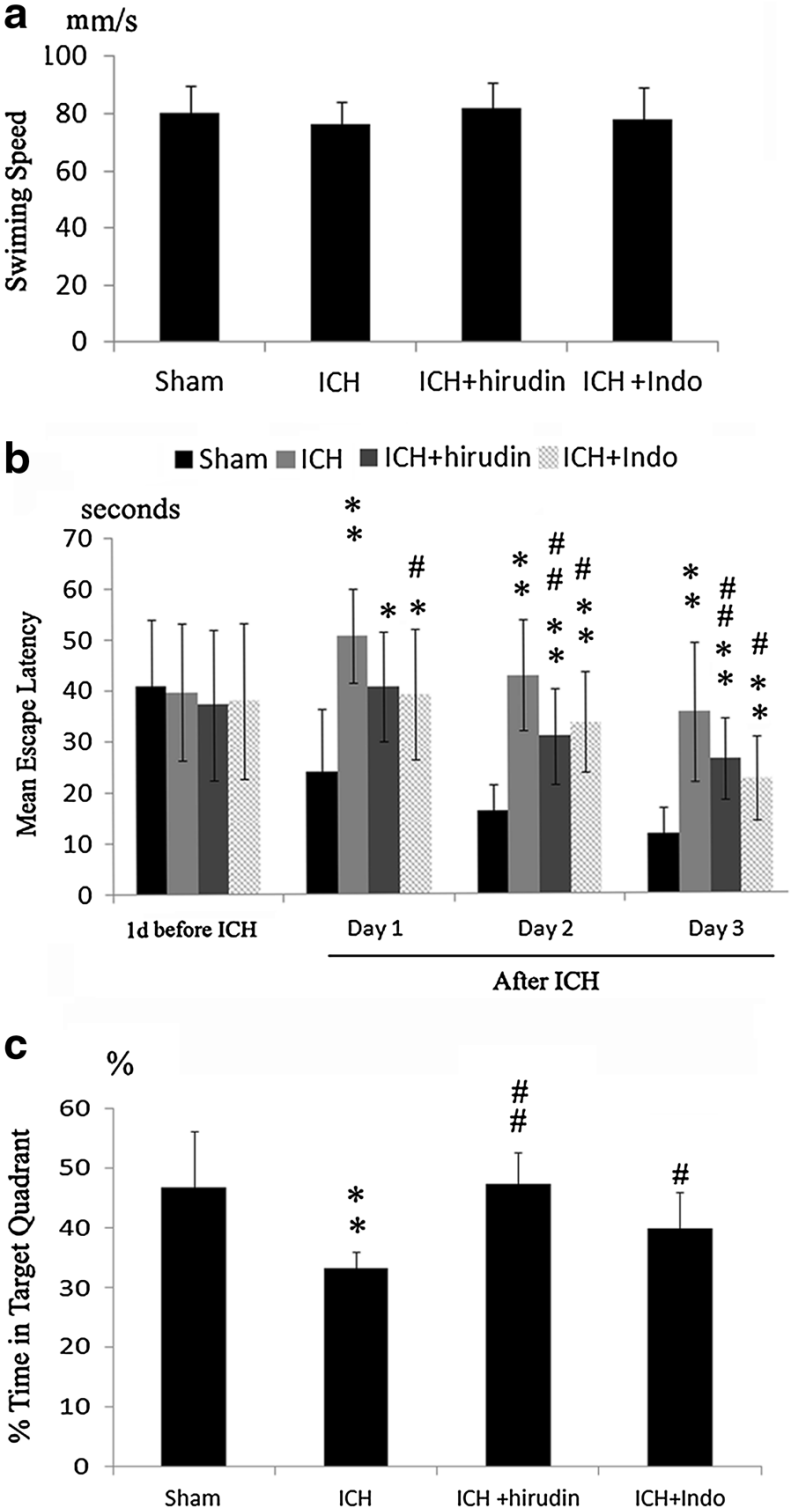
Effect of anti-inflammation treatment on the spatial memory ability of mice. **a** The representative swimming speeds of the mice at the first day after thrombin injection. **b** ICH decreased learning ability in mice. At 1–3 days after the thrombin injury, the escape latency in the ICH, ICH + hirudin and ICH + indo groups significantly increased compared to the shams. Hirudin decreased the escape latencies at day 2 and day 3 as compared with ICH group. Similarly, anti-inflammation treatment significantly improved the learning ability at day 1 to day 3 post-injury. There was no significant difference between hirudin and indomycin treatment groups at day 2 and day 3 post-injury. **c** The percentage of time spent in P quadrant significantly decreased in ICH group during the probe trial. Hirudin and indo treatment significantly increased the time spent in the P quadrant, compared with ICH group. *P < 0.05 as compared with sham; ^#^P < 0.05, ^##^P < 0.01 as compared with ICH group

In Morris water maze test, the speeds of mice did not change after thrombin injection or drug treatment (Fig. 5a). After 2 days adaptive training before the thrombin injection, the escape latencies between all animal groups were similar. In the next 3 days, the average latency to find the platform was 10–15 s in the sham mice. ICH significantly (P < 0.01) increased the latency to locate the platform, compared with the sham group (Fig. 5b). The latencies in the sham and ICH groups were 23.91 ± 12.53 s and 50.70 ± 9.20 s at day 1; 16.20 ± 5.28 s and 42.85 ± 10.85 s at day 2; 11.80 ± 4.99 s and 33.65 ± 13.61 s at day 3, respectively. At day 1, only indo treatment decreased the latency to locate the platform as compared with ICH group. At day 2 and day 3, both hirudin and indo treatments reduced the latency to locate the platform as compared with ICH group (Fig. 5b).

To evaluate memory retention, after 2 days rest, we performed a probe trial at the last MWM day. Mice were allowed to swim for 60 s in the pool with the escape platform removed. The percentage of time spent in the platform (quadrant P) quadrant was calculated for each animal. We found that the ICH group spent significantly (P < 0.01) less time in quadrant P (33.7 ± 4.53 %) than the sham group (46.72 ± 9.44 %) (Fig. 5c). Hirudin (47.29 ± 5.44 %) and indo (39.96 ± 6.08 %) treatments increased the time spent in quadrant P. Furthermore, there was no difference in the escape legacy or time in quadrant P between indo and hirudin groups (Fig. 5c).

## Discussion

In this study, we investigated the effects of intrastriatal thrombin injection induced neurotoxicity on the hippocampal neurogenesis and the spatial memory function in mice. We found that thrombin injection induced microglia/macrophages activation, decreased hippocampal neurogenesis and impaired cognition. Thrombin inhibitor and anti-inflammation drug treatments ameliorated these deficits.

It has been shown that there is a tight relationship between DG neurogenesis and the performance in MWM [27]. In our study, we found that the escape latency in Morris water maze was significantly increased in ICH mice, compared with shams. In addition, ICH mice spent significantly less time in the platform quadrant during the probe trial, suggesting the deficits in the memory retention. We also found that the hippocampal neurogenesis was significantly decreased in the ICH mice, compared with shams. Therefore, the spatial memory deficits in ICH mice might be due to the impairment of hippocampal neurogenesis. However, some studies demonstrate that water maze performance was unaffected after ablation of neurogenesis [28]. The discrepancy among the studies might be due to the differences in the design of MWM paradigms [29].

To date, there are very few reports regarding the effect of striatum ICH on spatial memory ability in adult mice. This may be due to the concern that sensorimotor deficits may interfere the spatial memory tests in the acute phase of ICH [30]. Hartman found that ICH caused learning impairments only in the first 8 weeks after ICH [31]. MacLellan et al. accessed the spatial memory function at 8 weeks after ICH to avoid confounding motor deficits with the spatial memory assessments. However, they did not find any cognitive deficits, which might be due to the timing of assessment [32]. In our study, we found that the cognitive deficit lasted at least for 5 days after the intrastriatal thrombin injection. During this period, the rotarod test (speed of 40 rpm, duration of 2 min) did not show any difference among all groups, suggesting no significant difference in fatigue among all groups. Because the Morris water maze test lasted for only 2 min, therefore, it is less likely that the animals’ performance in water maze was affected by the fatigue of limbs.

The beam walking assay is more sensitive than rotarod in determining motor deficit in mice [33]. In our study, a significant difference in walking beam test between sham and thrombin groups was observed only at 1 day after the thrombin injection. The times for all of the animals walking through the beam were similar at day 3 and day 5 after injury. However, subtle changes in motor function might be found between groups with more difficult paradigms, such as increasing the acceleration to 60 r.p.m or increasing the duration of rotarod test. In fact, if mice were kept running on the rotarod for 30 min, the ICH mice usually fell between 10–20 min whereas the sham mice could finish the 30-min test without falling (data not shown). Because the duration of Morris water maze test was a maximal 60 s and the swimming speed of all groups were not influenced by thrombin injection or therapeutic treatment (Fig. 5a), suggesting the 1U thrombin did not cause significant motor impairment at 3 and 5 days after the injury. This means the spatial memory deficit observed in water maze test was not due to the motor dysfunction after thrombin injection.

The role of thrombin after ICH remains controversial. High concentrations of thrombin induce neuronal damage in vitro, however, low concentrations of thrombin are neuroprotective against various insults including ischemia and oxidative stress [34]. Thrombin plays an important role in brain recovery after intracerebral hemorrhage [35], possibly via the initiation of neurogenesis [36] and angiogenesis [37]. In our experiments, 1U thrombin significantly decreased the DG neurogenesis and impaired the spatial memory ability in mice. Hence, 1U of thrombin has detrimental effects in our study. Hirudin, a thrombin inhibitor, specifically blocks the binding of thrombin to its protease-activated receptors (PARs). The inhibition of thrombin by hirudin significantly ameliorated the reductions in neurogenesis and improved the learning and memory in ICH mice. However, thrombin inhibitors might interfere in the clotting process; therefore it may not be a feasible treatment for ICH patients. Anti-inflammatory medication might be an alternative choice in ICH treatment.

Whether microglial activation has beneficial or detrimental effects remains controversial [38]. The resting microglia cells have been found to be neuroprotective in mild to moderate CNS disorders [39] and in hippocampal slice cultures [40]; whereas, microglia activation has detrimental effects in severe and chronic brain injuries [41]. In our study, anti-inflammatory drug treatment reduced the proliferation and/or activation of microglia/macrophages, increased hippocampal neurogenesis and rescued the spatial memory deficits after ICH. However, the underlying mechanisms need to be further explored.

There are some limitations in our study. Firstly, the current results only reflect the early neuronal changes after ICH. Future studies should examine neuronal changes weeks after ICH, since the cognitive deficits in patients often persist for a longer period of time. Secondly, activated microglia/macrophages usually damage neurogenesis by releasing proinflammatory cytokines, such as interleukin-6 (IL-6), interleukin-1b (IL-1b), and tumor necrosis factor- a (TNF-a), which in turn decrease cell survival and induce bias in neuronal differentiation [27]. However, which pro-inflammatory cytokines are involved in the damage of the DG neurogenesis need to be assessed. Finally, in addition to microglia/macrophages, astrocytes also play important roles in brain injuries, such as ICH and ischemia. More work needs to be done to define the specific roles played by the various inflammatory cells and cytokines in the brain.

## Conclusion

In summary, intrastriatal thrombin injection induced injury to DG neurogenesis and spatial memory function is partly mediated by inflammation, which was characterized by the activation of CD68 positive microglia/macrophages cells. Our study also revealed a potential role for anti-inflammation drugs in the regulation of activated microglia/macrophages, adult hippocampal neurogenesis, and spatial memory function after the intrastriatal thrombin injury.

## Abbreviations

BrdU: 5-bromo-2-deoxyuridine
DG: dentate gyrus
ICH: intracerebral hemorrhage
IHC: immunohistochemistry
MWM: Morris water maze
NSC: neural stem cell
SAH: subarachnoid hemorrhage
SGZ: subgranular zone
SVZ: subventricular zone
